# Imaging Features of Knuckle Pads

**DOI:** 10.5334/jbr-btr.1096

**Published:** 2016-07-12

**Authors:** Alexander De Keersmaeker, Filip Vanhoenacker

**Affiliations:** 1 University of Antwerp, BE; 2 AZ Sint-Maarten and University (Hospital) Antwerp/Ghent, BE

**Keywords:** knuckle pads, boxer’s nodules, MCP joint, PIP joint, MRI, US

A 50-year-old orthopaedic surgeon presented with painless well circumscribed soft tissue nodules at the dorsal aspect of the hand at the metacarpophalangeal (MCP) joint 4 and less pronounced at the MCP joint 2 (Figure 1, arrows). The nodules were slowly enlarging over a period of one year, without a history of an acute preceding trauma. They were asymptomatic. To reassure the patient, further imaging was performed.

**Figure 1:**
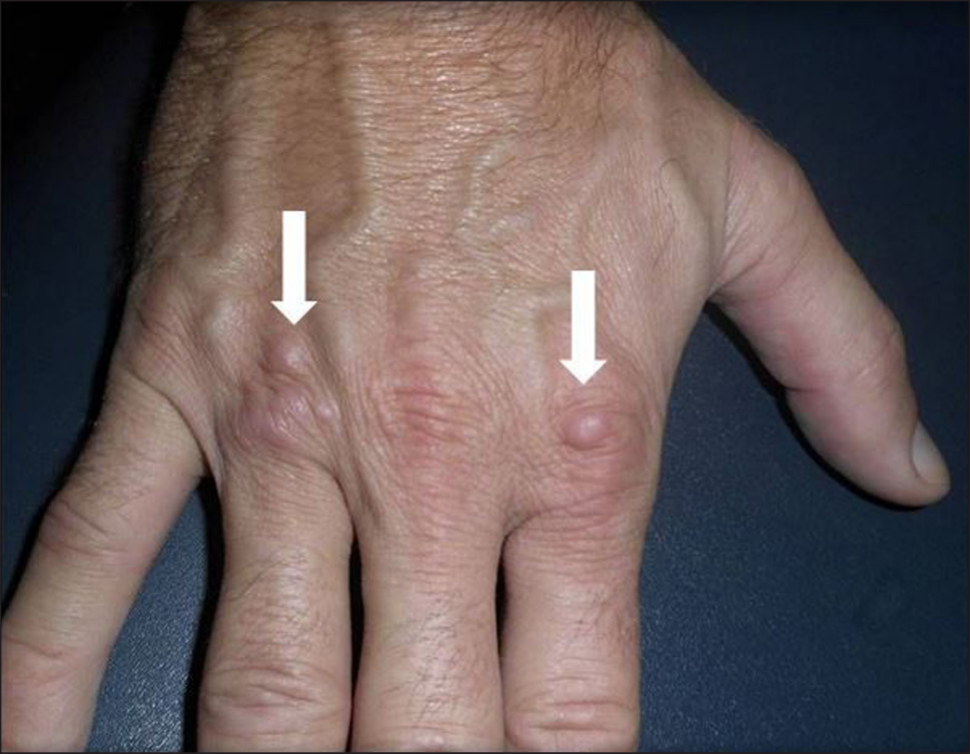


Ultrasound (US) showed two flat dome shaped hypoechogenic lesions at the subcutaneous aspect of MCP joints 2 and 4 (Figure 2, arrows).

**Figure 2:**
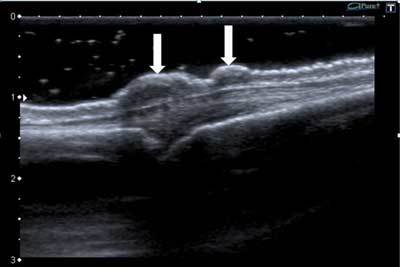


Axial T1-weighted image (WI) showed a hypointense nodule at the subcutaneous aspect of the extensor tendon of the fourth MCP joint. A subtler lesion was seen at of the extensor side of second MCP joint (Figure 3A, arrows).

**Figure 3:**
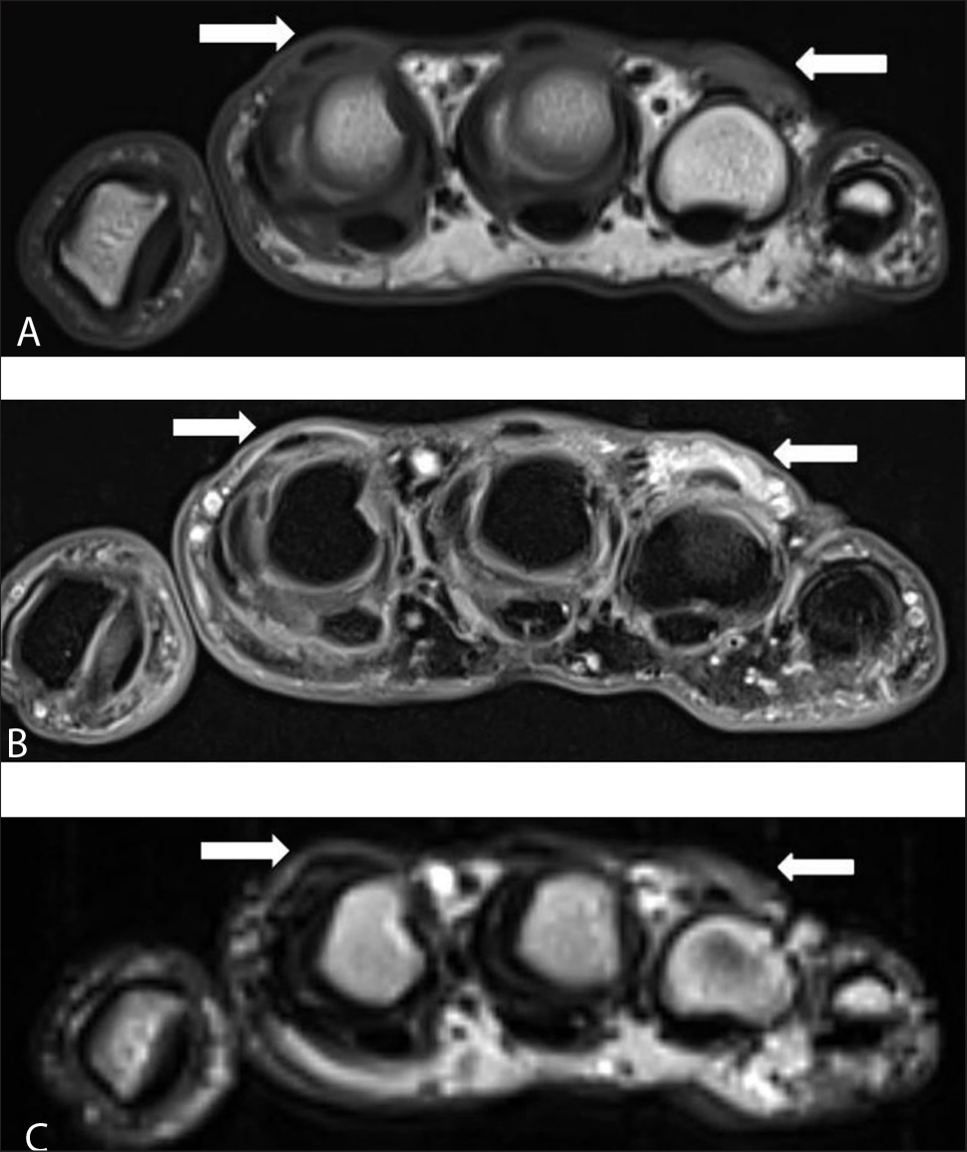


On axial fat suppressed (FS) T2-WI, the lesions were of relatively high signal compared to the surrounding tissues (Figure 3B, arrows), whereas the nodules were of intermediate signal on T2-WI without fat suppression (Figure 3C, arrows).

Based on the combination of the clinical findings, the typical location and the imaging findings, the diagnosis of knuckle pads was made. Furthermore a wait-and-see policy was recommended.

## Comment

Knuckle pads are benign subcutaneous fibrotic plaques. Clinically, these lesions present as slowly enlarging, painless and mobile nodules, typically located at the extensor site of the proximal interphalangeal (PIP) and MCP joints. They are often the consequence of repetitive trauma. In this regard, they are often seen in boxers, hence the designation as *boxer’s nodules*. However, any profession or activity causing chronic friction at the MCP and PIP joints may result in the development of knuckle pads. There is an association with other fibrotic disorders, such as palmar (Dupuytren’s disease) and plantar fibromatosis (Ledderhose’s disease). In the paediatric population, knuckle pads have been described as being idiopathic [1].

On ultrasound, knuckle pads present as subcutaneous hypoechogenic nodular thickenings localized over the dorsal aspect of the MCP or PIP joint. They have a typical dome shape with irregular borders. The lesions are not compressible with the ultrasound transducer. On Doppler ultrasound, vascularization is absent or restricted to the periphery of the lesion. The adjacent soft tissues, joints and tendons are not involved [1].

MRI has only been rarely described in the literature [2]. The location of the lesion is the most useful clue to the diagnosis. The low to intermediate signal intensity of the lesion on T1-WI and T2-WI may correspond to fibrosis, although the signal of fibrosis may vary according to the age of fibrosis. Old fibrosis is typically of low signal on both pulse sequences, whereas young fibrosis may be of higher signal, particularly when fat suppressed T2-WI images are used.

The differential diagnosis includes Bouchard and Heberden’s nodes of osteoarthritis, rheumatoid nodules, gouty tophi and giant cell tumor of the tendon sheaths [1]. Plain radiographs may be helpful in the differential diagnosis with osteoarthritis, gout and rheumatoid arthritis. A giant cell tumor, which is more often located at the flexor tendons, is of low signal on all MR pulse sequences and may show blooming artifact on gradient echo imaging.

There is no effective treatment for knuckle pads and watchful waiting is usually recommended.

In conclusion, although the diagnosis of knuckle pads can be made solely on the clinical presentation and typical location at the MCP and PIP joint, the radiologist has to be aware of this disease and its imaging features in order to avoid misdiagnosis, unnecessary biopsy and potentially harmful treatment.
